# Microbiome-Responsive Hydrogels: From Biological Cues to Smart Biomaterials

**DOI:** 10.3390/pharmaceutics18030284

**Published:** 2026-02-24

**Authors:** Rajesh Vadlapatla, Amir Nasrolahi Shirazi, Ajoy Koomer, Judy Weng, Matthew Ernest Ghilarducci, Alai Qudus, Keykavous Parang

**Affiliations:** 1 Department of Pharmaceutical Sciences, College of Pharmacy, Marshall B. Ketchum University, 2575 Yorba Linda Blvd., Fullerton, CA 92831, USA; ashirazi@ketchum.edu (A.N.S.); akoomer@ketchum.edu (A.K.); jweng@ketchum.edu (J.W.); matthewghilarducci.cop28@ketchum.edu (M.E.G.); alaiqudus.cop26@ketchum.edu (A.Q.); 2 Center for Targeted Drug Delivery, Department of Biomedical and Pharmaceutical Sciences, Chapman University School of Pharmacy, Harry and Diane Rinker Health Science Campus, 9401 Jeronimo Rd, Irvine, CA 92618, USA; parang@chapman.edu

**Keywords:** microbiome-responsive hydrogels, stimuli-responsive hydrogels, enzyme-responsive materials, microbial metabolites, smart biomaterials, controlled drug delivery, biological triggers

## Abstract

**Background:** Stimuli-responsive hydrogels (SRHs) are smart polymeric materials that undergo reversible physicochemical changes in response to abiotic cues and externally applied fields, enabling applications in drug delivery, wound healing, and tissue engineering. However, they exhibit limited biological specificity and do not adequately reflect the dynamic, disease-relevant complexity of native tissue microenvironments. Microbe-colonized tissues display distinctive biochemical features driven, shaped by microbial metabolism, including localized pH gradients, short-chain fatty acid production, secretion of quorum-sensing molecules, biofilm formation, and expression of specialized enzymes. These endogenous, spatiotemporally regulated signals are closely linked to host physiology and pathology but remain underutilized in hydrogel design. This review aims to highlight microbiome-responsive hydrogels (MRHs) as a strategy to address this gap. **Methods:** This study summarizes current engineering approaches, key microbial stimuli, and emerging biomedical applications of MRHs, with emphasis on translational and regulatory challenges. **Results:** Microbiome-responsive hydrogels (MRHs) address this gap by leveraging microbial metabolic and biochemical cues to induce swelling, degradation, drug release, antibacterial activity, or structural transformation. By directly coupling to microbe-derived stimuli, MRHs offer improved physiological relevance, enhanced local specificity, and new opportunities for precision therapy targeting disease-associated microbial niches. **Conclusions:** Despite their promise, MRHs remain an early and fragmented field, lacking standardized biological triggers, material design frameworks, and performance evaluation strategies. This review summarizes current engineering approaches, key microbial stimuli, and emerging biomedical applications, with emphasis on translational and regulatory challenges, positioning MRHs as an underexplored platform for next-generation smart biomaterials.

## 1. Introduction

Hydrogels are three-dimensional (3D), crosslinked polymeric networks capable of absorbing and retaining large amounts of water or biological fluids while preserving their structural integrity [1,2]. These networks can be constituted through covalent crosslinks or through an extensive number of noncovalent interactions, including physical entanglements, hydrogen bonding, hydrophobic interactions, supramolecular assembly, electrostatic forces, and coordination interactions [3]. Natural polymers such as polypeptides, polysaccharides, and DNA, and synthetic polymers, like polyacrylamide and poly(vinyl alcohol), have been extensively investigated in regard to hydrogel preparation due to their chemical versatility and tunable structure–property relationships [1,3].

Hydrogels might be tuned to provide a wide range of physicochemical and functional properties, including mechanical strength, elasticity, toughness, stretchability, adhesiveness, self-healing behavior, and shape-memory characteristics relevant to pharmaceutical formulation and drug delivery performance, via modification of polymer composition and crosslinking mechanism [4]. Because of their flexible physicochemical properties and structural and functional similarity to the extracellular matrix, hydrogels have emerged as versatile platforms for controlled drug delivery, tissue engineering, biosensing, and biomedical device design, and have consequently achieved substantial market penetration across the personal care, biomedical, and pharmaceutical sectors [5,6,7,8,9].

Based on composition, origin, and response behavior, hydrogels can be broadly classified according to source (e.g., natural, synthetic, or hybrid), crosslinking type (e.g., physical (reversible) or chemical (covalent)), charge characteristics (e.g., neutral, ionic, amphoteric), degradability (biodegradable or nondegradable), and responsiveness to external or internal stimuli (e.g., passive or stimuli-responsive (“smart”)) [1,10]. This classification framework is particularly relevant in pharmaceutics, as it informs material selection, formulation strategy, and anticipated in vivo performance. Among these categories, SRHs have received considerable attention for their ability to dynamically modulate drug release and material behavior in response to environmental cues, prompting focused discussion of their design principles and physicochemical responsiveness.

### 1.1. Stimuli-Responsive Hydrogels: Design Strategies, Clinical Translation, and Biological Limitations

SRHs, also termed “smart” hydrogels, represent a second generation of hydrogels depending mainly on abiotic physicochemical stimuli, including pH, temperature, ionic strength, redox environment, mechanical stress, and externally applied physical stimuli such as light, magnetic fields, and electrical signals [11,12,13,14]. These smart polymeric networks are engineered to experience reversible physicochemical transitions in response to these environmental stimuli [11,14]. Within the drug delivery context, such responsiveness has been leveraged to improve site-specific delivery, prolong half-life, and reduce systemic exposure. The clinical feasibility of this approach is illustrated by several U.S. Food and Drug Administration (FDA)-approved products. UGN-101 (“Jelmyto™”, UroGen Pharma, Princeton, NJ, USA), a thermo-responsive hydrogel formulation containing mitomycin C, is used to treat low-grade upper urothelial carcinoma; it remains liquid at room temperature and rapidly gels at body temperature, enabling localized drug delivery within the urinary tract [15]. In addition, Vantas^®^ (Endo Pharmaceuticals, Malvern, PA, USA), a hydrogel depot composed of crosslinked polymers, provides sustained release of histrelin acetate for palliative therapy in advanced prostate cancer [15]. Similarly, ELIGARD^®^ (Tolmar, Fort Collins, CO, USA), an in situ-forming polymeric gel, forms a subcutaneous depot after injection to enable controlled release of leuprolide acetate for prostate cancer treatment [15].

SRHs are commonly classified according to the nature of the stimulus that induces changes in their swelling behavior, structure, or mechanical properties. One of the earliest and most studied thermo-responsive hydrogels exhibits reversible phase changes at the lower critical solution temperature (LCST): the polymer chains remain hydrated and expanded below the LCST but collapse when heated owing to enhanced hydrophobic interactions [16,17]. This swift and reversible response has allowed for applications in injectable systems, tissue-engineering scaffolds, and on-demand drug delivery [4,18].

pH-responsive hydrogels are another broad-spectrum approach that uses ionizable functional groups like carboxylates, amines, and sulfonates, whose protonation state changes in pH, affecting electrostatic repulsion and network swelling [14,19]. For instance, poly(acrylic acid) will swell at neutral to alkaline pHs, whereas chitosan-based hydrogels will expand preferentially in acidic environments due to the protonation of amines [19,20]. Such structures have been highly used for cancer treatment, wound healing, and tissue engineering [8,20,21].

Other SRHs respond to additional physicochemical cues, including ionic strength, redox conditions, light, or electromagnetic fields. Ionic strength and osmotic-responsive hydrogels incorporate charged moieties that swell or contract in response to changes in ion concentration, valence, or osmotic gradients, making them useful for wound management, biosensing, and controlled delivery, although specificity remains limited in complex biological environments [22,23,24]. Redox-responsive hydrogels exploit cleavable or rearrangeable linkages, including disulfide bonds, thioketals, and boronate esters, enabling degradation or softening in oxidative or reductive environments relevant to inflammation, cancer, and intracellular delivery [25,26,27]. Photo-responsive hydrogels incorporate photodegradable or photoisomerizable groups that translate light exposure into reversible or irreversible network changes through bond cleavage or conformational switching [28,29,30,31,32,33]. Finally, electro- and magneto-responsive hydrogels respond to applied fields by altering swelling or inducing deformation, supporting applications in drug delivery and other biomedical technologies [34,35].

Conventional SRHs, while versatile and extensively developed, are predominantly engineered to respond to simplified, abiotic physicochemical cues that only partially represent the complexity of living systems. In microbiome-rich tissues, however, local biochemical conditions are continuously reshaped by resident and pathogenic microorganisms through enzyme secretion, metabolite production, redox modulation, and intercellular signaling. These dynamic, biologically driven processes are poorly represented by static changes in pH, temperature, or externally applied stimuli. This mismatch between material responsiveness and biological reality has motivated the emergence of a new class of biomaterials, microbiome-responsive hydrogels (MRHs) that leverage endogenous microbial signals to achieve greater specificity, contextual responsiveness, and disease-relevant activation [36].

Though key to hydrogel science, most conventional SRHs use non-endogenous triggers, limiting their use in microbiome-rich tissues and their biological relevance. The development of MRHs represents a move towards endogenous, biologically informed activation strategies.

Table 1 summarizes the key technical design features and translational considerations that distinguish conventional stimuli-responsive hydrogels from MRHs.

### 1.2. Translational Challenges of SRHs

Conventional SRHs have shown great potential in preclinical studies; however, their translation into clinical use is hindered by material limitations and the complexity of physiological and regulatory environments. This section highlights the key translational gaps, their underlying causes, and their implications for clinical viability.

#### 1.2.1. Biocompatibility and Safety

One fundamental challenge associated with SRHs is not only the biocompatibility of an intact hydrogel network but also the degradation byproducts. Most responsive hydrogels are based on synthetic monomers (e.g., acrylamides, methacrylates, polyethylene glycol derivatives), which can degrade under physiological conditions into small molecules or oligomers [37,38,39,40,41]. The toxicity, immunogenicity, or clearance profiles of these degradation products remain poorly characterized. For example, acrylamide monomers, a degradation product of a widely used polymer network, are known to possess neurotoxicity, nephrotoxicity, or even carcinogenic potential [42,43]. Even biocompatible hydrogels may induce immune responses, depending on the manufacturing process and fabrication methods, such as crosslinker chemistry, additives (including nanoparticles and plasticizers), and surface properties [44].

#### 1.2.2. Physiological Complexity

SRHs are mostly designed and validated in simplified and controlled in vitro conditions that fail to model the complexity of physiological environments [45]. In vivo, heterogeneity in ionic strength, unique protein diversity, variable fluid flow, diverse enzymatic activity, and host immune responses mediate the hydrogel’s performance [44,46,47]. Swelling kinetics, mechanical response, and degradation modes or pathways of swelling can be dramatically influenced by the aforementioned variables, consequently creating unexpected material responses and decreased predictability [44,45,46].

#### 1.2.3. Mechanical Stability

Hydrogels prepared via reversible, noncovalent interactions are attractive as they are flexible and easily deliverable. Nevertheless, their intrinsic mechanical weakness and limited long-term stability limit their clinical applications [48]. Cyclic swelling, redox fluctuations, or enzymatic stimulation can gradually destabilize the network, leading to lower mechanical strength and durability, and these systems are less suitable in applications where long-lasting structural support is required [48]. Consequently, finding a compromise between dynamic responsiveness and structural robustness remains a major challenge for the next design hurdles.

#### 1.2.4. Drug Loading and Release Kinetics

SRHs have been investigated as controlled release platforms for various small-molecule, protein/peptide therapeutics, and nucleic acids by researchers in recent years. Nevertheless, loading capacity is a big limitation. The hydrogel networks are predominantly water-swollen, thus restricting the stable incorporation of hydrophobic drugs and large biomolecules. Hydrophobic drugs may aggregate or phase-separate, while proteins and nucleic acids can diffuse out prematurely or become ineffective [49]. Several hydrogels exhibit non-ideal release kinetics, characterized by an initial burst followed by slow or incomplete release, which compromises sustained therapeutic delivery and increases the risk of localized toxicity or inadequate long-term dose control. Additionally, in vivo release behavior is often poorly predicted by in vitro release profiles due to microenvironmental variability, leading to unpredictable dosing and reduced therapeutic safety margins [50,51]. Collectively, these constraints complicate the development of reliable and reproducible SRH-based dosage forms.

The clinical experience with UGN-101 (Jelmyto™), a thermo-responsive hydrogel formulation approved for the treatment of low-grade upper tract urothelial carcinoma (LG-UTUC), illustrates several of these challenges. Although UGN-101 has demonstrated clinical efficacy, its use is associated with notable safety and tolerability concerns. Ureteral stenosis, resulting in impaired urinary flow, was the most frequently reported treatment-emergent adverse event in the pivotal OLYMPUS trial, with risk increasing alongside repeated instillations and often necessitating additional interventions such as ureteral stent placement [52]. In addition, patients may experience urinary tract infections, hematuria, flank or abdominal pain, fatigue, and renal complications, while bone marrow suppression has also been reported and requires clinical monitoring [53]. Meta-analytical evidence further indicates that adverse events occur in a substantial proportion of treated patients, with a subset discontinuing therapy due to intolerance, underscoring the translational limitations associated with current SRH-based delivery systems [54].

#### 1.2.5. Manufacturing, Scale-Up, and Regulatory Considerations

Clinical translation of SRHs is further hindered by challenges related to manufacturing scalability, material reproducibility, and regulatory uncertainty. Many formulations require complex polymerization chemistries and specialized monomers, leading to batch-to-batch variability and inconsistent performance [55,56]. Minor changes during synthesis, sterilization, or storage can significantly alter the material. In parallel, unclear regulatory pathways for responsive and hybrid biomaterials add uncertainty, increasing development time, cost, and translational risk [55,56].

Together, these challenges contribute to a significant translational gap. Although proof-of-concept studies of SRHs have been shown in vitro and in small-animal models, few have advanced to clinical trials or regulatory approval. This gap reflects the mismatch between controlled laboratory conditions and complex human physiology, as well as unresolved manufacturing, safety, and regulatory barriers [57,58].

### 1.3. The Rationale for MRHS

**Figure 1:**
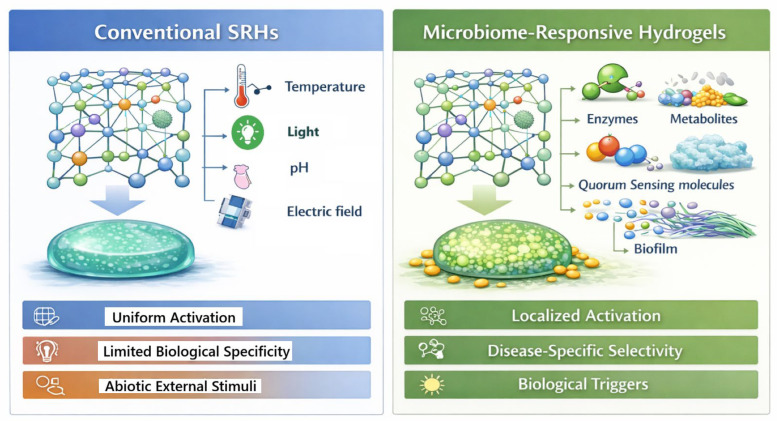
Conventional SRHs and MRHs: Conceptual comparison. The conventional SRHs in general are sensitive to physicochemical stimuli (temperature, pH, electric fields) with homogenized and nonspecific activation. Conversely, MRHs are activated by microbial signals, which are enzymes, metabolites, quorum-sensing molecules, and biofilms, enabling localized, disease-targeted, and biologically precise responses.

In light of the shortcomings of conventional SRHs, there is growing interest in these so-called materials that are responsive to biologically relevant, endogenous signals, namely those generated by resident or pathogenic microbiota (Figure 1) [59,60]. Within tissues densely colonized by microorganisms, such as in the gut, skin, oral cavity, and urogenital tract, microbial activity will generate defined microenvironments with microenvironmental signatures such as enzymatic activity, metabolic byproducts, quorum-sensing molecules, and redox or pH gradients that mirror states of disease [61]. Adding sensitivity to these stimuli enables the activation of the hydrogels preferentially in pathological scenarios such as infection, dysbiosis, or inflammation, rather than to general physicochemical changes [60]. This biologically sensitive methodology reduces external stimuli dependency, allows for temporally and spatially focused response, and has potential clinical applications through enhanced specificity and patient compliance [36,62]. Such factors make MRHs a biologically aligned and translationally effective advance of smart biomaterials [62,63].

### 1.4. Endogenous Biological Triggers Beyond Abiotic Stimuli

The majority of conventional SRHs are based on exogenous or abiotic physicochemical triggers that restrict their capability to authentically capture the dynamic biochemical mechanisms that control biological environments [63]. Microbes, like bacteria, fungi, parasites, and viruses, have colonized various body sites and together influence the local biochemical landscape through enzyme secretion, metabolite production, and the generation of redox-active species. Such microbially driven biochemical dynamics are not represented by the static changes in pH or temperature. Consequently, conventional SRHs often fail to respond to these complex physiological triggers, driving the development of microbiome-responsive materials [60].

These biologically responsive or microbiome-responsive materials, rather than relying on artificially generated cues, exploit the molecular signatures inherent in microbial ecosystems. Such MRHs could provide superior specificity, dynamic responsiveness, and target-specific activation that is tightly coupled to disease-associated microenvironments [64].

While conventional SRHs are designed to operate within biologically inert frameworks, MRHs sense and respond to interact with tissues as active, microbially dynamic biochemical environments [65]. Pathogenic bacteria secretions, metabolites generated through microbial fermentation, redox-active species produced during respiration, and quorum-sensing molecules involved in microbial communication may all function as endogenous activation triggers [36,66].

### 1.5. Evolution of Microbiome as a Source of Biologically Relevant Stimuli

Enzyme-responsive hydrogels (ERHs) were initially demonstrated to undergo selective degradation in response to microbial enzymes, such as β-lactamases. This concept has since evolved to include metabolite-responsive systems that sense fermentation byproducts, such as short-chain fatty acids (SCFAs) and ammonia [67]. Collectively, these approaches define a new class of biologically integrated hydrogels that overcome key limitations of conventional SRHs.

Together, these advances position MRHs as a mechanistically distinct and biologically integrated class of smart biomaterials. To rationally design such systems, a detailed understanding of the microbiome-derived biochemical cues that define disease-relevant microenvironments is essential, as discussed in the following section.

## 2. The Microbiome as a Source of Biologically Relevant Stimuli

**Figure 2:**
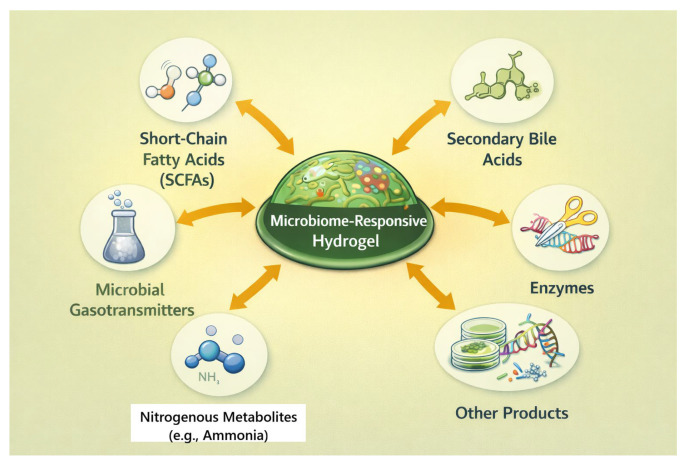
Microbial metabolite gradients and hydrogel activation. This schematic illustrates the concept of MRHs, in which material behavior is governed by biologically derived cues rather than generic physicochemical stimuli. Microbial metabolites, enzymes, and signaling molecules generated within colonized tissues interact with the hydrogel network to induce localized swelling, degradation, or therapeutic release. By selectively responding to disease-associated microbial activity, such hydrogels enable context-aware activation, improved specificity, and closer alignment with the native biological environment.

Human-associated microbial communities generate a rich array of biochemical and physicochemical signals, metabolites, enzymes, communication molecules, and biofilm-associated microenvironments that vary dynamically in space and time [68,69] (Figure 2). These cues, obtained from microbes, typically correlate with normal physiological or pathological states (e.g., infection, dysbiosis, and inflammation) and are highly appealing as endogenous motivators of the next generation of MRHs [36]. The result of these microbial consortia is a diversity of biochemical outputs, metabolites, enzymes, signaling molecules, and biofilm-associated substances, resulting in unique and disease-relevant microenvironments [68,69]. This section explores these different categories of microbial cues and their potential as triggers for next-generation MRHs [36,69].

### 2.1. Microbial Metabolites as Hydrogel Triggers

Microbial metabolism transforms nutrients and host-derived substrates into a wide variety of small molecules [70,71]. In regions such as the gut, skin, oral cavity, and wound sites, where dense or pathogenic microbial growth occurs, these metabolites accumulate locally and can generate sharp chemical gradients [71,72,73]. Relevant biochemical triggers for hydrogel activation include SCFAs, microbial gasotransmitters, nitrogenous metabolites such as ammonia, secondary bile acids, and other products of microbial metabolism [70,72,74]. Table 2 represents microbial metabolites that can be exploited as endogenous triggers for smart hydrogel activation, along with their potential applications.

As shown in Table 2, various stimuli can elicit MRH responses. More details about nature and mechanisms are elaborated below.

#### 2.1.1. Short-Chain Fatty Acids (SCFAs) and Organic Acids

Dietary fibers are fermented, particularly in the colon, by fermentative anaerobic bacteria into SCFAs (acetate, propionate, and butyrate) [70]. These metabolites reach high local concentrations (tens to hundreds of mM) and impact host physiology, including immune modulation, epithelial barrier integrity, and metabolism [70,75]. Such volatile fatty acids containing fewer than six carbons may lower local pH or alter ionic strength and protonation state, and the presence of ionizable groups (carboxylates and amines, for example) that can influence the behavior of hydrogels may be ideal triggers for hydrogels built with ionizable groups in response to pH and/or ionic changes [14]. SCFAs may also activate host signaling pathways through G-protein-coupled receptors (e.g., GPR41 and GPR43), thereby affecting immune responses systematically [70,76].

#### 2.1.2. Gasotransmitters and Reductive Metabolites (e.g., H_2_S, NO, Sulfides)

In addition to fermentation, microbial communities engage in diverse metabolic pathways that generate reductive metabolites and gasotransmitters, including hydrogen sulfide (H_2_S), nitric oxide (NO), and other redox-active species [72,77]. Altered gut sulfur metabolism during disease can drive localized accumulation of H_2_S [77,78]. The microbial-generated H_2_S or NO may stimulate redox-sensitive linkages (such as disulfide bonds) and lead to polymeric degradation or network softening [72].

#### 2.1.3. Bile Acid-Derived and Other Microbial Catabolites

Within the gastrointestinal tract, microbial metabolism converts host-derived primary bile acids into secondary bile acids that exert substantial effects on host physiology and microbial community structure [74,79]. Hydrogels incorporating bile acid-responsive or amphiphilic components could exploit these metabolites to trigger conformational rearrangements or solubility changes in response to these metabolites [79,80]. This conformational rearrangement or change in solubility allows spatially targeted drug release or material transformation in the distal small intestine and colon [72,80].

#### 2.1.4. Nitrogenous Waste and Ammonia Production

Urease-producing microbes, such as *Proteus* and *Klebsiella* species, generate urease enzymes that convert urea into ammonia [81,82]. This leads to an increase in local pH and alters the chemical microenvironment at infection sites, such as chronic wounds, dental plaques, and the urinary tract. These microbe-induced alkaline areas are different from healthy tissue environments. pH-sensitive hydrogels that respond to alkaline environments could exploit these conditions to provide site-specific therapeutic release or antimicrobial activity [14,81].

### 2.2. Microbial Enzymes and Enzyme-Triggered Hydrogels

**Figure 3:**
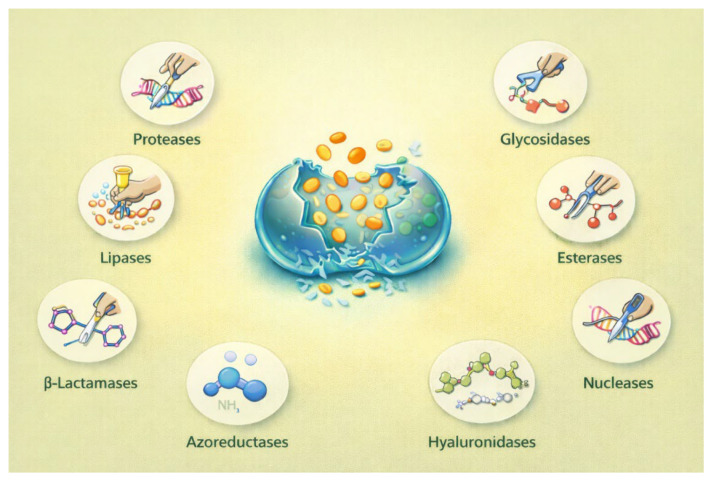
Enzyme-triggered hydrogel degradation and cargo release. This illustration shows a hydrogel matrix that undergoes localized degradation in the presence of microbially secreted enzymes, including proteases, lipases, glycosidases, esterases, β-lactamases, azoreductases, hyaluronidases, and nucleases. Enzyme-mediated cleavage of labile linkers within the network enables controlled structural breakdown and targeted release of encapsulated therapeutic cargo in enzyme-rich, microbe-associated environments.

In addition to metabolite-based signals, microbial enzymes represent a particular class of biological triggers for responsive materials. A wide range of bacteria and fungi secrete enzymes (e.g., proteases, lipases, glycosidases, esterases, β-lactamases, azoreductases, hyaluronidases, and nucleases), either continuously or in response to environmental conditions such as quorum sensing or biofilm formation [83]. These enzymatic processes are often closely linked with virulence and tissue invasion and offer trustworthy biochemical indicators of active microbial colonization [83,84,85]. Inclusion of enzyme-cleavable linkers or labile motifs (e.g., peptide sequences, polysaccharide backbones, azo bonds, or ester linkages) in hydrogel networks can facilitate the engineering of ERHs. These ERHs experience preferential degradation or property alteration in enzymatically enriched microenvironments (Figure 3) [34,84]. This approach brings improved specificity while minimizing unintended effects in healthy or sterile tissues.

The literature describes ERHs composed of natural polymer networks, e.g., hyaluronic acid and chitosan, functionalized with peptide crosslinkers susceptible to matrix metalloproteinases, bacterial proteases, or fungal hydrolases [34,86]. These ERHs maintain desirable characteristics such as swelling and porosity while supporting enzyme-specific degradation and therapeutic release [34,85,86].

In wound healing, a dual pH- and enzyme-responsive hydrogel composed of gelatin methacryloyl (GelMA) and an oxidized alginate–antibiotic conjugate exhibited accelerated degradation and antibiotic release in acidic, enzyme-rich, infected wounds compared to neutral, enzyme-poor environments [87]. This selective activation enhanced bacterial clearance and supported tissue regeneration while minimizing systemic exposure [87].

ERHs offer several advantages, including high specificity toward particular microbes or pathological states, which enables targeted and localized activation [34,84]. Such spatially regulated response is useful for reducing off-target effects, and biodegradable natural polymers, such as chitosan or gelatin, contribute to biocompatibility [14,84]. While it has these advantages, ERHs also encounter significant limitations. Microbial enzymes are typically contained in biofilms or host extracellular matrices [83], which reduces available hydrogel linkers and the efficiency of activation. Moreover, the expression of the above enzymes differs greatly with the microbial species and conditions, so that it is difficult to achieve consistent and predictable activation [34,83]. Finally, the premature degradation by host-derived or environmental enzymes can limit specificity, underscoring the importance of assessing in vivo stability in order to design and develop hydrogels [14,84].

### 2.3. Microbial Communication Molecules and Biofilm-Derived Signals

Microbial communities exert their behavior coordination to quorum-sensing (QS) molecules in addition to metabolism and enzyme secretion [88]. Such low-molecular-weight, diffusible signals control density-dependent microbial behaviors—the synthesis of biofilms, virulence expression, and dispersal [88,89]. Some common QS molecules are N-acyl homoserine lactones (AHLs) of many Gram-negative bacteria, autoinducing peptides (AIPs) of Gram-positive bacteria, and diffusible signal factors (DSFs) of some species [88,89]. Biofilm formation produces unique microenvironments of characteristic extracellular polymeric substances (EPSs) composed of polysaccharides, proteins, extracellular DNA, and concentrated enzymes/metabolites [83,89]. Biofilms change local pH, redox, ionic strength, rheology, and diffusion characteristics together forming a microenvironment separate from that in planktonic cultures or host tissues [83,90].

#### 2.3.1. QS Molecules as Potential Hydrogel Triggers

QS molecules are appealing activators for hydrogel-stimulating signals, because they generally accumulate to measurable levels only after microbial populations reach critical densities, for example, during biofilm formation [88,89]. Such signals are indicative of active colonization or pathogenic behavior rather than passive microbial residence. As early-stage signaling molecules, QS signals can also act as warning biomarkers and provide early diagnostics or therapeutic measures [88,89]. In principle, hydrogels could be shaped to be capable of integrating molecular recognition elements (e.g., aptamers, molecularly imprinted polymers, or receptor-derived binding domains) capable of recognizing QS molecules, selectively binding them to induce conformational changes leading to swelling or deswelling or therapeutic release [89,91]. Thus, to be able to deliver targeted antimicrobial intervention while maintaining commensal populations and minimizing resistance development, these systems should respond only to virulence-associated signals [88,89,91].

#### 2.3.2. Biofilm-Responsive Hydrogels via EPS and Microenvironment Sensing

The EPS matrix produced by biofilms modifies local diffusion, ion content, hydration, and redox balance [83]. Hydrogels can be designed to sense these changes through EPS-cleavable linkers, redox- or ion-responsive motifs that detect biofilm metabolism, or surface-binding domains that interact with EPS components to drive swelling or contraction [73].

In addition, biofilm-responsive hydrogels can potentially perform therapeutic release and mechanically disrupt biofilms. Hydrogels that undergo swelling, stiffness changes, or shape-memory transitions may physically disrupt EPS, improve antimicrobial penetration, or support immune clearance [36,73]. Recent reviews on hydrogel-based biosensors and therapeutics highlight the potential of these multifunctional platforms in infection control and microbiome engineering [36].

### 2.4. Microbiome-Induced Physicochemical Shifts: pH, Redox, Osmotic, and Ionic Changes

Microbial growth and metabolism frequently induce local physicochemical changes within tissues [36]. For example, lactic acid production by oral or dental biofilms lowers pH, whereas ammonia production by urease-active bacteria can raise local pH in wounds or infections. Anaerobic microbial communities may generate redox gradients, and biofilm aggregation can modify local osmotic pressure and ionic strength [36].

Hydrogels that incorporate pH-sensitive, redox-sensitive, or ionic-strength-sensitive moieties, such as ionizable groups, disulfide bonds, phenylboronic esters, or dynamic noncovalent crosslinks, can thus serve as indirect sensors of microbial activity [19,92]. Indeed, in wound-healing applications, ROS or ERHs have been used to release antimicrobials, scavenge oxidative stress, and deliver anti-inflammatory agents in response to infection-associated biochemical changes [93].

While these approaches do not directly detect microbial species, they exploit the metabolic consequences of microbial colonization, offering a pragmatic route toward microbiome-responsive materials even in the absence of highly specific molecular triggers [19,92].

### 2.5. Summary

Human tissue-hosted microbial systems generate a wide range of biochemistry-related signals: bio-metabolites, enzymes, quorum-sensing compounds, redox-active species, and other chemical-biochemistry signals. Furthermore, they promote physicochemically heterogeneous microenvironments via biofilm formation and metabolism. These microbe-derived stimuli provide a biologically relevant and disease-associated source of endogenous cues for responsive hydrogels. Utilizing these cues, MRHs could attain high specificity as well as spatial and temporal precision and adaptive capabilities, thereby overcoming the drawbacks of conventional SRHs that are based on generic abiotic stimuli. To achieve that potential, a strong understanding of microbial ecology, metabolite kinetics, enzyme secretion profiles, diffusion constraints, and host–microbe–material interactions is necessary. Early experimental studies, including ERHs, pH/redox-sensitive wound dressings, and microbiome-modulating scaffolds, have shown these materials to be feasible. MRHs research may serve as a new class of smart biomaterials closely combined with living microbial ecosystems and have an enormous impact on drug delivery, infection control, tissue regeneration, diagnostics, and precision medicine.

## 3. Design Strategies and Material Architecture for MRHs

**Figure 4:**
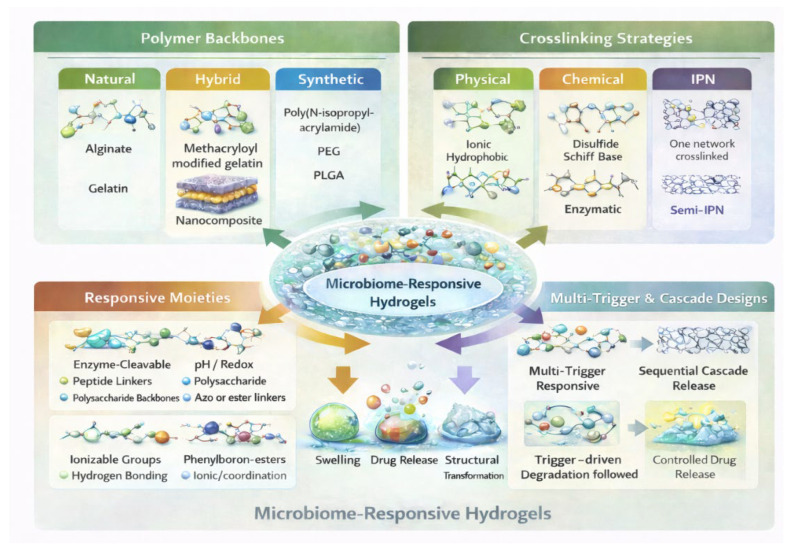
Systematic summary of modular design components leveraged to fabricate state-of-the-art hydrogel systems, encompassing polymer backbone choice (natural, synthetic, hybrid), crosslinking approaches (physical, chemical, IPN, and semi-IPN), design of responsive moieties (enzyme-cleavable, pH/redox, and noncovalent interactions), and multi-trigger and cascade architectures to achieve programmable structural and functional responses.

The construction of MRHs demands a synergistic alignment of polymer chemistry, network arrangement, and stimulus-responsive elements for a selective, resilient, and reversible response to microbial stimuli [6]. Unlike traditional SRHs, MRHs must function in highly heterogeneous, dynamic, and biologically complex microenvironments where metabolites, enzymes, redox gradients, pH changes, quorum-sensing molecules, and biofilm-driven physicochemical changes coexist [83,94]. Through fundamental design strategies, material architectures, and examples, this section demonstrates the rational engineering of MRHs for biomedical applications [6,94] (Figure 4).

Based on a polymer backbone and the appropriate chemical crosslinking agents, the structural requirements for these compounds are defined.

### 3.1. Polymer Based on Origin and Source

Natural polymers: These macromolecules consist of nucleic acids, alginate, chitosan, gelatin, hyaluronic acid, collagen, agarose, dextran, and cellulose derivatives, which are bio- or naturally derived materials [95,96,97]. Their intrinsic biocompatibility, biodegradability, hydrophilicity, low immunogenicity, and bioactive functionalities (e.g., cell adhesion patterns) make them excellent materials for biomedical uses. Moreover, they can be engineered through peptide or small-molecule linkers to behave with specific enzyme- or metabolite-responsive properties. Some of their drawbacks [98,99] are poor mechanical strength, high viscosity, poor thermal stability, and high internal degradation.

Synthetic polymers: Synthetic polymers such as poly lactic-co-glycolic acid (PLGA), polylactic acid (PLA), polyethylene glycol (PEG), polyvinyl alcohol (PVA), polyacrylamide, poly(N-isopropylacrylamide) (PNIPAM), polymethacrylates and their co-polymers are fully synthetic polymers [95,100]. These polymers offer accurate and precise control over physical and chemical properties, e.g., crosslinking density, mechanical strength, swelling behavior, low friction coefficient, and reproducibility [101,102,103,104,105,106]. Lack of endogenous factors and, therefore, biological inertness is the weakness of synthetic polymer(s) [107]. In addition, they can be toxic or immunogenic, particularly upon degradation. These types of polymers require careful selection and safety assessment of all polymers and degradation products used.

Hybrid (semi-synthetic/composite) hydrogels: These are a combination of natural and synthetic polymers or chemically modified natural polymers (e.g., methacryloyl-modified gelatin), or prepared by the incorporation of reinforcing fillers, nanoparticles, or fibers [107]. They aimed to integrate the favorable bioactivity of natural polymers with the tunability and mechanical robustness of synthetic ones. Nanocomposite hydrogels (e.g., polymer + clay or graphene components; hyaluronic acid-PEG is a semi-synthetic hydrogel) are good examples of this class.

In terms of design principles, hybrid systems that integrate natural and synthetic polymers are often used to balance biological responsiveness, mechanical stability, and tunability, which is particularly important for wound dressings, gut-targeted delivery, or implantable devices.

### 3.2. Polymer Based on Crosslinking Type

Physically crosslinked (noncovalent) hydrogels: These hydrogels have transient junctions and are formed by noncovalent interactions such as hydrogen bonding, ionic interactions (electrostatic), hydrophobic associations, van der Waals interactions, entanglements, or crystallite domains. Examples of noncovalent hydrogels include gelatin and alginate. Gels formed by physical crosslinking are generally reversible (shear-thinning, self-healing, stimulus-responsive) and highly responsive to environmental stimuli, but often exhibit limited mechanical robustness and rapid degradation [108]. These hydrogels are suitable for wound healing because cyclic bacterial colonization may need repeated antimicrobial release [65].

Chemically crosslinked hydrogels: These hydrogels use chemical crosslinking agents that undergo chemical reactions to form a strong crosslinked network with permanent junctions. Chemical agents commonly used for such crosslinkages include glutaraldehyde, formaldehyde, and genipin [109,110]. These crosslinkages are formed through covalent bonds (e.g., via radical polymerization, enzyme-mediated reactions, Michael addition, and Schiff base linkages). Examples of noncovalent hydrogels include chitosan and pectin. These networks yield hydrogels that are more robust, stable, and less prone to deformation and enzymatic degradation, but their degradability and self-healing properties must be optimized for optimal performance. Embedded enzyme-labile linkers form permanent networks ideal for drug depots where predictable degradation triggers controlled release [44].

Semi-interpenetrating network (semi-IPN): Hydrogels in which the crosslinked polymer network is physically interlaced with one or more non-crosslinked polymer chains and have no covalent bonds acting between them [1,2]. This configuration combines the mechanical performance of the crosslinked network with the functional flexibility of the linear polymer, resulting in improved swelling, controlled drug release, and stimuli-responsive behavior of the semi-IPN [111,112]. Semi-IPNs have been widely studied for biomedical and pharmaceutical applications due to their tunable properties and superior performance compared to simple homopolymeric or copolymeric hydrogels [1,111].

Interpenetrating polymer networks (IPNs): These hydrogels are formed by two or more independently crosslinked and interlaced polymer networks without covalent bonds between them. In this combination of networks, an enhanced material is formed instead of one hydrogel. IPNs can be created using different methods, such as sequential or simultaneous polymerization of the two networks. These IPNs can provide a compromise between stiffness and toughness, modulate swelling, and fuse multifunctional features in a modular manner [113].

### 3.3. Molecular and Network-Level Design Principles for Microbiome Responsiveness

Enzyme-cleavable linkers: Peptide motifs sensitive to bacterial proteases or small-molecule linkers cleavable by specific microbial enzymes (e.g., β-lactamases) can be incorporated in network crosslinks to enable targeted degradation and cargo release [114,115]. The β-lactamase-responsive hydrogel exemplifies this strategy, with degradation and cargo release observed only in the presence of β-lactamase-producing bacteria; non-responding controls remained stable under the same conditions [115,116,117].

pH/Redox-sensitive chemical groups: Ionizable side groups (e.g., carboxyl, amine), phenylboronic esters, disulfide bonds, or redox-labile moieties permit sensitivity to microbial metabolite-induced pH or redox shifts [14,92]. Previously, ROS-sensitive hydrogels have been used to treat microbial infections and release antibiotics in response to high ROS levels induced by bacterial metabolism and inflammatory oxidative stress [93,118].

Dynamic noncovalent interactions: Hydrogels built from reversible interactions (e.g., hydrogen bonding, ionic interactions, metal coordination, host–guest chemistry) can undergo reversible transitions [119,120]. These may be particularly useful when hydrogels need to repeatedly respond to fluctuating microbial cues while maintaining structural integrity [119]. Reviews of peptide- and protein-based responsive hydrogels highlight these design strategies [120,121,122].

Multi-trigger and cascade-responsive designs: Microbial niches seldom produce a single stimulus, which is why MRHs can provide multiple responses by using multiple cues, such as combinations of enzymatic activity, pH changes, and redox changes, as well as biofilm-associated EPS signals [14,123]. This multi-input structure increases selectivity and reduces unexpected activation in diverse biological systems [6,14]. The reviews of injectable and multi-SRHs have highlighted this as an important route for clinical translation [6,124]. Moreover, cascade designs, in which an initial microbial signal initiates a first hydrogel response that leads to a secondary response (e.g., enzyme-triggered degradation followed by drug release, ROS release, or immunomodulator activation), potentially enable more advanced control of dosing, timing, and spatial delivery [120,123].

## 4. Biomedical Applications of MRHs

MRHs offer novel possibilities in many therapeutic and diagnostic uses. The following are those critical aspects in which these materials have shown to be promising or, in particular, demonstrate great promise in several important domains.

### 4.1. Infection-Responsive Antimicrobial Delivery and Wound Healing

Among the most immediate applications of MRHs is in infection-responsive antimicrobial therapy. Enzyme-cleavable or ROS-sensitive linkers have been fabricated to deliver antibiotics, nitric oxide (NO), or antimicrobial agents in hydrogels only in the presence of bacterial or metabolic activity, thereby avoiding inappropriate exposure and decreasing the chances of inducing resistance [93,115,125]. For example, a β-lactamase-responsive hydrogel delivered encapsulated nanoparticle cargo only when β-lactamase-producing bacteria were present and remained unresponsive in their absence of these organisms [114,115]. A self-acting hydrogel dressing was developed using bacterial metabolites that initiated a series of NO release, biofilm breakdown, antibacterial activity, and wound healing in an infected wound model [115].

In MRSA-infected wound models, spatiotemporally sequenced dual release of ROS-responsive antibiotics and anti-inflammatory agents was obtained, leading to rapid antimicrobial release followed by persistent anti-inflammatory response [93,118]. Recently reported bacteria-responsive hydrogel with a polydopamine-based surface design exhibited resist–kill–release with ongoing antimicrobial and antifouling effects and extended wound protection [126]. These samples illustrate how MRHs are adaptively tailored to an infection microenvironment to maximize efficacy in pathogen elimination while decreasing injury and sustaining healthy tissue and microbiota [93,114,125].

### 4.2. Microbiome Modulation and Tissue Regeneration

In addition to antimicrobial delivery, hydrogels have been developed to modulate microbial communities and support tissue regeneration, particularly in microbiome-rich tissues such as skin, gut, or mucosal surfaces [127,128,129]. Recently, some hydrogels which were developed for the selective administration of probiotics, prebiotics, bacteriocins, immunomodulators, or growth factors have been shown to be applied in a controlled way to support healthy microbial regulation, re-establish a proper balance, inhibit pathogens, and enhance immune tolerance or tissue repair [36,127,128]. Using microbiome-responsive triggers, these hydrogels can release their payloads only when dysbiosis, such as infection or inflammation, occurs, enabling precision microbiota engineering and minimizing off-target effects [128,129].

### 4.3. Targeted Delivery in Complex Microenvironments (Gut, Oral, Mucosal, Biofilm-Rich Tissues)

MRHs hold particular promise for targeted drug delivery in tissues where microbial communities shape local biochemistry, such as the gut, oral cavity, vagina, or chronic wounds [6,14,83]. These regions often exhibit gradients in pH, redox state, enzymatic activity, or biofilm density. For instance, pH- or redox-responsive hydrogels could release drugs preferentially in regions with microbial dysbiosis [14,83]. Enzyme-responsive systems could selectively activate in areas rich in microbial proteases or β-lactamases [6,14,115]. Biofilm-responsive designs might disrupt pathogenic biofilms or deliver agents specifically when biofilm formation begins, enabling early intervention. Such targeted delivery, informed by microbial microenvironmental cues, could improve therapeutic efficacy, reduce systemic toxicity, and preserve beneficial commensal populations [6,14,83].

### 4.4. Biosensing, Diagnostics, and Smart Implants

Moreover, MRHs show promise as biosensing or diagnostic platforms [130,131,132]. Integrated molecular recognition elements (e.g., enzyme-reactive linkers, receptor–ligand pairs, aptamers, macrophage infectivity potentiator-like binding sites) within a hydrogel matrix can enable devices that sense microbial activity, metabolite production, or biofilm formation in situ [130,131]. These can be early signs of infection, dysbiosis, or microbial imbalance in implants, wounds, or mucosal surfaces [131,132]. Additionally, when combined with drug-loaded components, these hydrogels could act as smart implants, delivering therapeutics only in response to microbial stimuli or providing clinicians with feedback via quantifiable changes (e.g., swelling, degradation, colorimetric shifts) [120,132].

## 5. Critical Challenges and Translational Barriers

MRHs are still predominantly experimental, although advances have been made quickly, and proof-of-concept experiments are showing promise. However, several critical challenges must be met in order to achieve clinical usefulness:

### 5.1. Heterogeneity and Complexity of Microbial Environments

Microbial communities vary widely between tissues, individuals, and disease states [133,134]. Enzymes or metabolism of the same bacterial species can differ depending on growth conditions, nutrient availability, and the presence of microbial neighbors [134,135]. MRHs engineered for a single set of microbial cues may therefore be inadequate across different clinical contexts or patient populations. Designing materials that accommodate such variability, or identifying conserved microbial triggers, remains a significant challenge [133,135].

### 5.2. Lack of Standardization and Quantitative Characterization

Quantitative kinetics, dose–response relationships, reproducibility, and in vivo stability [6,136] are absent, despite qualitative responsiveness (e.g., gel degradation, drug release) being demonstrated in many studies. When characterization protocols are not standardized, it is challenging to compare systems and to translate clinical-grade materials [136,137]. Furthermore, environmental factors in vivo, including protein adsorption, immune factors, dynamic fluid flow, and tissue buffering, will alter the hydrogel response in comparison to in vitro buffer systems [6,137]. Therefore, performance in simple in vitro assays may poorly predict in vivo behavior.

### 5.3. Biocompatibility, Safety, and Degradation Products

Hydrogels intended for clinical use need to be not only biocompatible but also degrade to products that are safe [138,139]. Synthetic polymers can degrade into small molecules with unclear toxicity (e.g., acrylamide-based fragments), and dynamic degradation in response to microbial triggers can generate unpredictable byproducts [139,140]. Previous reviews have reported that degradation toxicity is frequently neglected [139]. Immunogenicity, foreign-body reactions, or unintended disruption of commensal microbiota are also significant risks for microbiome-responsive applications [138,140].

### 5.4. Scaling, Manufacturing, and Regulatory Hurdles

Moving away from bench-level demonstration to scalable, reproducible production within good manufacturing practice (GMP) conditions is a significant challenge [136,141]. Since MRHs are adaptive and rely on biological signals, regulatory bodies may recognize them as combination products (device + biologic) or even as advanced therapy medicinal products, thereby imposing more stringent regulatory requirements. Therefore, it is challenging to establish batch-to-batch consistency, stability, and shelf-life compared to traditional inert biomaterials [136,141,142].

### 5.5. Limited Mechanistic Understanding and Predictive Design Frameworks

Polymer chemistry and materials science contribute tools for designing responsive networks, yet the integration of polymer physics with intricate biological stimuli (microbial metabolism, enzymatic kinetics, redox gradients, diffusion, and fluid flow) remains poorly defined [6,143]. These designs are mostly empirical, with little mechanistic or computational modeling that accounts for these factors [6,144]. This is an obstacle to systematic hydrogel development for differing microbial settings or patient populations [6,144].

## 6. Future Directions and Opportunities

MRHs have characterized a new direction in terms of biological materials that have gone hand in hand with those of microbial ecosystems. However, beyond physicochemical triggers, MRHs may transcend the main translational limits attributed to traditional SRHs and may contribute to precision and context-sensitive therapeutic interventions. Achieving this potential will require the closure integration of materials science with microbiome biology, computational modeling, and translational engineering.

Because microbial environments are rarely defined by a single isolated cue, hydrogels capable of integrating multiple, unrelated microbial cues, such as enzymatic activity in combination with localized pH, redox, or biofilm-associated signals, represent a critical advance in MRH design. The inherent complexity of microbial microenvironments, which are rarely driven by a single stimulus, makes multi-trigger responsiveness, enabling higher specificity and operational robustness.

Hierarchical polymer designs, such as double-network or interpenetrating systems, separate mechanical strength from biological responsiveness, and dynamic crosslinking allows reversible activation in changing microbial environments. Together, these strategies better mimic natural biological sensing and reduce unintended activation [6,14,120].

Designing hydrogels that respond to the microbiome itself is a crucial step moving forward. Once the microorganisms’ metabolism, signaling, and community structure are understood, the behavior of the hydrogel can be further shaped to respond to biologically relevant cues rather than nonspecific physicochemical sources. Using databases such as metagenomics and proteomics has enabled the identification of enzymes, metabolites, and quorum-sensing molecules associated with disease-related microbial states [145,146,147]. These insights enable the development of hydrogels for applications such as dysbiotic gut disorders, chronic wound infections, and pathogen-dominated metabolic niches.

Progress in this field also depends on quantitative and predictive characterization frameworks. The absence of standardized benchmarks for responsiveness, reversibility, and long-term stability has limited both reproducibility and regulatory translation. Future work should be focused on physiologically significant testing systems, which are better representative of biological reality by integrating proteins, extracellular matrix elements, resident microorganisms, and evolving environments (fluid flow, buffering, etc.) to enhance the quality of results. Integrating such models and high-throughput and real-time analysis will be critical to characterize dose–response relationships, activation kinetics, and degradation and failure modes in highly diverse microbial environments [14,136,137].

Experimental analysis will be supplemented by computational modeling and biophysical simulation. Multi-scale computational models that link polymer network behavior with microbial system biology enable the prediction of MRH performance under physiologically relevant conditions. Such a systems-level approach minimizes reliance on the traditional trial-and-error method and enables rational formulation optimization for targeted tissues and disease states [148,149,150,151]. Over time, these computational tools may facilitate the development of adaptive or personalized MRH platforms.

Safety, biocompatibility, and regulatory strategies are other areas that require careful attention for clinical adoption of MRH. Beyond short-term cytocompatibility, these materials must be evaluated for their effects on immune responses, microbiome perturbation, degradation kinetics, and long-term exposure [138,139,152]. Selecting materials with known safety profiles and predictable degradation products can reduce regulatory delays, and early communication with regulatory agencies will also clarify approval pathways and establish expectations for testing and manufacturing quality.

Another fascinating aspect is the development of hybrid therapeutic–diagnostic (theranostic) MRHs. By coupling molecular recognition elements with stimuli-responsive release and reporting functionalities, MRHs can behave as autonomous, closed-loop systems [130,153,154]. Such platforms could enable smart implants, infection-monitoring wound dressings, or gut-targeted systems that deliver therapeutics only when pathogenic activity is detected, thereby minimizing unnecessary drug exposure and reducing selective pressure for resistance [130,154,155].

MRHs remain mostly at the research and preclinical stage. As of today, there are no truly stimuli-responsive MRHs commercially available on the market. Existing hydrogel products, whether for wound care, drug delivery, or tissue engineering, do not yet incorporate programmable responses specifically tuned to microbial composition or activity. Personalized, microbiome-responsive therapies represent a natural extension of the MRH platform. Variability in microbial composition and activity across patients warrants customizable material responses (e.g., tailored wound dressings or gut-targeting treatments). Adaptive MRH platforms capable of tuning trigger thresholds or updating response profiles in real time could support precision medicine approaches in wound care, gastrointestinal disorders, and mucosal infections.

Finally, successful translation depends on scale-up and manufacturing innovation. Modular designs that decouple the base hydrogel from interchangeable responsive elements could ease scale-up and regulatory review. Robust sterilization, storage, and stability protocols must be established to preserve functionality, while GMP-compliant production with stringent quality control is essential for clinical adoption.

In summary, the future of MRHs lies in the integration of multi-trigger material design, microbiome-informed personalization, quantitative characterization, computational modeling, and translational disciplines. Aligning material responses with the dynamic nature of microbial ecosystems positions MRHs as promising adaptive platforms for precision therapy, real-time monitoring, and broad biomedical impact.

## 7. Conclusions

MRHs are a promising development from conventional stimuli-responsive biomaterials. These materials have the potential for biological specificity, spatial and temporal precision, and adaptive behavior in complex tissues via endogenous microbial signals, metabolites, enzymes, redox or pH shifts, communication molecules, and biofilm-specific microenvironments. Initial experiments show that infection-responsive antimicrobial delivery, wound healing, and manipulation of the microbiome are feasible. However, realizing the full potential of this approach requires addressing critical challenges, including the heterogeneity of microbial environments, the lack of quantitative and reproducible characterization, biocompatibility and safety concerns, manufacturing and regulatory barriers, and a scarcity of predictive design frameworks. Addressing these challenges will demand interdisciplinary collaboration across materials science, microbiome biology, computational modeling, and clinical translation, but the reward could be a new generation of “smart” biomaterials tightly integrated with the living microbial ecosystems of the human body.

## Figures and Tables

**Table 1:** Technical and translational differences between SRHs and MRHs.

Dimension	Stimuli-Responsive Hydrogels (SRHs)	Microbiome-Responsive Hydrogels (MRHs)
Primary trigger source	Abiotic or externally applied cues (pH, temperature, redox, light, magnetic/electric fields)	Endogenous, microbe-derived biochemical and metabolic cues
Nature of stimulus	Physicochemical and largely generic	Biological and context-dependent (microbial enzymes, metabolites, signaling molecules)
Biological specificity	Low to moderate; the same stimulus may occur in healthy and diseased tissues	High; triggers are linked to microbial composition and activity at a given site
Spatiotemporal control	Often coarse and externally imposed or systemically present	Intrinsic and localized to microbial niches; dynamically reflects microbial activity
Mechanistic activation	Polymer phase transitions (swelling, collapse, bond cleavage) driven by environmental parameters	Material responses driven by microbial metabolism, enzymatic reactions, or signaling pathways
Physiological relevance	Limited representation of complex host–microbe interactions	Directly aligned with host–microbiome microenvironment and disease biology
Therapeutic targeting potential	Broad or tissue-level targeting	Precision targeting of disease-associated microbial communities or infected/inflamed niches
Robustness and predictability	Generally high and reproducible across models	More variable due to inter-individual and temporal microbiome heterogeneity
Design complexity	Relatively well-established material chemistries and architectures	Requires integration of microbiology, enzymology, and material design
Translational maturity	More advanced, including clinically validated systems	Early-stage and fragmented; limited standardization and validation frameworks
Key translational challenge	Achieving adequate site specificity and controlled activation in vivo	Managing microbiome variability, safety, and regulatory qualification of biological triggers

**Table 2:** Microbial metabolite classes and microenvironmental cues as endogenous triggers for microbiome-responsive hydrogels.

Microbial	Representative Metabolites/Signals	Typical Microbial Sources/Sites	Microenvironmental Effect	Hydrogel Activation Mechanism	Potential Biomedical Applications
SCFAs	Acetate, propionate, butyrate [70]	Gut microbiota (*Bacteroides*, *Firmicutes*); chronic wounds [70]	Local pH reduction; osmotic changes	pH-responsive swelling/deswelling; ionizable polymer transitions	Colon-targeted drug delivery; inflammation-responsive release
Microbial gasotransmitters	Hydrogen sulfide (H_2_S), nitric oxide (NO), carbon dioxide (CO_2_) [72]	Anaerobic gut bacteria; infected or ischemic wounds [72]	Redox modulation; gas accumulation	Redox-sensitive linkers; gas-responsive degradation	Infection-responsive drug release; wound healing
Nitrogenous metabolites	Ammonia, amines [71]	Oral biofilms; skin; urogenital tract [71,73]	Local alkalinization; chemical stress	pH-triggered network collapse or accelerated degradation	Oral disease treatment; antimicrobial delivery
Secondary bile acids	Deoxycholic acid, lithocholic acid [74]	Intestinal microbiota-mediated bile metabolism [74]	Formation of hydrophobic microdomains; membrane perturbation	Hydrophobic interaction-driven network reorganization	Gut-specific drug release; metabolic disease intervention
Biofilm-associated metabolite gradients	Mixed organic acids, alcohols, indoles [71,73]	Biofilms in wounds, gut, and medical-device-associated infections [73]	Sharp spatial chemical gradients; localized hypoxia	Gradient-dependent spatial activation or erosion	Biofilm-targeted therapy; site-specific delivery

## Data Availability

No new data were created or analyzed in this study.

## Abbreviations

The following abbreviations are used in this manuscript:

AHL	N-acyl homoserine lactones
AIP	Autoinducing peptides
ATMP	Advanced therapy medicinal products
DSF	Diffusible signal factors
EPS	Extracellular polymeric substances
ERH	Enzyme-responsive hydrogels
GelMA	Gelatin meth acryloyl
GMP	Good manufacturing practice
GPR41	G-protein-coupled receptor 41
GPR43	G-protein-coupled receptor 43
H_2_S	Hydrogen sulfide
IPN	Interpenetrating polymer networks
LCST	Lower critical solution temperature
MIP	Molecularly imprinted polymers
MRH	Microbiome-responsive hydrogels
NO	Nitric oxide
PEG	Polyethylene glycol
PLA	Polylactic acid
PLGA	Poly(lactic-co-glycolic acid)
PNIPAM	Poly(N-isopropylacrylamide)
PVA	Polyvinyl alcohol
QS	Quorum sensing
ROS	Reactive oxygen species
SCFA	Short-chain fatty acids
semi-IPN	Semi-interpenetrating polymer networks
SRH	Stimuli-responsive hydrogels
